# Case Report: Cerebellopontine angle metastatic adenocarcinoma with high-grade fetal morphology and no identifiable pulmonary primary on initial CT

**DOI:** 10.3389/fonc.2026.1942218

**Published:** 2026-10-27

**Authors:** Xiaowen Liu, Chengxin Yan

**Affiliations:** Department of Medical Imaging, The Second Affiliated Hospital of Shandong First Medical University, Tai’an, China

**Keywords:** brain metastasis, case report, cerebellopontine angle, high-grade fetal morphology, imaging-pathology correlation, metastatic adenocarcinoma

## Abstract

**Background:**

Adenocarcinoma with high-grade fetal morphology can create diagnostic uncertainty when it presents intracranially without an identifiable systemic primary.

**Case presentation:**

A 61-year-old man presented with gait instability for more than 1 month, dizziness, and left-sided hearing loss. CT and MRI showed a left cerebellopontine angle (CPA)-region mass with heterogeneous avid enhancement, fourth-ventricle compression, and suspected brainstem invasion, without internal auditory canal enlargement. On contrast-enhanced T1-weighted MRI, the lesion measured 37.6 × 24.7 × 35.9 mm (left-to-right [transverse] × anteroposterior × craniocaudal). Initial thoracic CT identified no pulmonary lesion. Subtotal resection was performed because of dense adherence to cranial nerves and the brainstem. Histology showed complex fetal-type glandular and papillary architecture, supranuclear and subnuclear vacuoles, high-grade atypia, and necrosis. The integrated diagnosis was metastatic adenocarcinoma with high-grade fetal morphology, favoring pulmonary origin; however, no pulmonary primary was identifiable on initial thoracic CT or confirmed pathologically. The diagnosis of pulmonary high-grade fetal adenocarcinoma (HG-FLAC) was therefore not established. Postoperative dysphagia and facial nerve dysfunction developed. Systemic staging remained incomplete; molecular testing and PD-L1 assessment were not performed. Remaining paraffin-embedded tissue was insufficient for additional immunohistochemical studies. No systemic anticancer therapy or radiotherapy was initiated after tertiary-center consultation, and detailed treatment-decision records were unavailable. The patient died 3 months after surgery; the immediate cause was undocumented.

**Conclusion:**

This case illustrates an atypical CPA presentation of adenocarcinoma with high-grade fetal morphology. Pulmonary origin was favored but unconfirmed. Diagnostic terminology must reflect this uncertainty, and the short survival cannot establish HG-FLAC-specific prognosis.

## 1 Introduction

Fetal adenocarcinoma of the lung resembles developing fetal lung tissue. Its low-grade and high-grade forms differ in morphology, immunophenotype, and clinical behavior. Pulmonary high-grade fetal adenocarcinoma (HG-FLAC) typically exhibits marked atypia, necrosis, often absent morules, and sometimes divergent neuroendocrine differentiation (1–4). Evidence remains limited by its rarity.

The cerebellopontine angle (CPA) is a common location for extra-axial intracranial masses, particularly vestibular schwannoma, meningioma, and epidermoid cyst. CPA metastases may mimic these lesions, including in patients without known malignancy (5, 6). We report a CPA-region adenocarcinoma with high-grade fetal morphology and no pulmonary primary identified on initial CT. The diagnostic lesson concerns both recognition of an atypical CPA malignancy and the limits of assigning its primary site.

## 2 Case description

### 2.1 Patient information and clinical findings

A 61-year-old man presented with gait instability for more than 1 month, dizziness, and left-sided hearing loss. He had a 60 pack-year smoking history, occasional alcohol consumption, a previous cerebral infarction, and controlled grade 1 hypertension. No previous malignancy or oncological intervention was documented. Family, psychosocial, and germline genetic histories were unavailable. The case is de-identified.

Finger-to-nose testing demonstrated dysmetria. Facial motor function and swallowing were clinically normal before surgery; hearing loss was confirmed clinically. The remaining detailed neurological examination was incompletely documented. Serum carcinoembryonic antigen was 13.30 ng/mL (reference interval, 0-5), and alpha-fetoprotein (AFP) was 4.946 ng/mL (0–10). Other measured markers, including CA125, CA19-9, total and free prostate-specific antigen, squamous cell carcinoma antigen, CA242, CA72-4, neuron-specific enolase, and cytokeratin 19 fragment, were within laboratory reference intervals. **Table 1** summarizes the clinical course.

**Table 1:** Timeline of clinical presentation, investigations, treatment, and outcome.

Time point	Event	Findings	Management or interpretation
>1 month before admission	Symptom onset	Gait instability, dizziness, and left-sided hearing loss.	Evaluation for a posterior fossa/CPA lesion.
At admission	Clinical evaluation	Dysmetria; facial function and swallowing normal; hearing loss confirmed. CEA 13.30 ng/mL; serum AFP within reference interval.	Neurological and imaging assessment.
Initial imaging	Brain CT/MRI	37.6 × 24.7 × 35.9 mm (left-to-right [transverse] × anteroposterior × craniocaudal) left CPA-region mass; heterogeneous enhancement; mild edema; fourth-ventricle compression; suspected brainstem invasion; no IAC enlargement.	Differential included schwannoma, meningioma, epidermoid, and metastasis.
Initial staging	Thoracic CT	No identifiable pulmonary lesion or suspicious thoracic mass/lymphadenopathy.	Pulmonary primary not demonstrated; staging incomplete.
Surgery	Retrosigmoid resection	Firm hypervascular tumor adherent to cranial nerves and brainstem.	Subtotal resection with intentional residual tumor.
Postoperative pathology	Histology/IHC	High-grade fetal morphology; diffuse pan-CK; focal TTF-1/SALL4; partial synaptophysin; focal weak CD56; Ki-67 approximately 80%.	Metastatic adenocarcinoma with high-grade fetal morphology, favoring pulmonary origin. No lung primary on initial CT or pathological confirmation; pulmonary HG-FLAC not established.
Early postoperative period	Neurological outcome	Dysphagia with coughing/choking on liquids; impaired cheek puffing and lagophthalmos.	Facial and swallowing dysfunction; no documented ECOG/KPS score.
After tertiary consultation	Oncological care	No systemic anticancer therapy or radiotherapy; detailed multidisciplinary recommendations, eligibility assessments, and preference/refusal documentation unavailable.	Best supportive care locally.
3 months after surgery	Follow-up	Death; immediate cause undocumented.	Subtype-specific prognosis and direct cause of death cannot be inferred.

AFP, alpha-fetoprotein; CEA, carcinoembryonic antigen; CPA, cerebellopontine angle; CT, computed tomography; ECOG, Eastern Cooperative Oncology Group; HG-FLAC, high-grade fetal adenocarcinoma of the lung; IAC, internal auditory canal; IHC, immunohistochemistry; KPS, Karnofsky performance status; MRI, magnetic resonance imaging.

### 2.2 Diagnostic assessment

#### 2.2.1 Neuroimaging and systemic evaluation

Brain CT demonstrated a slightly hyperdense left CPA-region mass with mass effect on the brainstem and cerebellum. The internal auditory canals (IACs) were not enlarged. No definite adjacent petrous bone erosion, hyperostosis, or tumor calcification was established.

MRI showed a lobulated mass measuring approximately 37.6 × 24.7 × 35.9 mm (left-to-right [transverse] × anteroposterior × craniocaudal), centered in the left CPA/cisternal region. Measurements were obtained on contrast-enhanced T1-weighted MRI: the left-to-right and anteroposterior dimensions were measured on axial images, and the craniocaudal dimension was measured on a sagittal image. It compressed the adjacent brainstem, cerebellum, and fourth ventricle. The brainstem interface raised suspicion of invasion, but definite brainstem parenchymal invasion was not established. The lesion had an extra-axial-like location; a purely extra-axial compartment could not be confidently established because of suspected involvement at the adjacent parenchymal interface. No definite intracanalicular extension, broad dural attachment, or dural tail was identified. The lesion was closely related to the facial-vestibulocochlear nerve complex. Its relationship to the lower cranial nerves was established mainly intraoperatively. Separate involvement of the cerebellar vermis and the precise site of origin within the CPA cistern were not established from the available documentation.

The mass was isointense on T1-weighted imaging and predominantly isointense on T2-weighted imaging, with internal linear mildly hyperintense areas. FLAIR showed mixed iso- to mildly hyperintense signal and mild adjacent edema. There was no definite diffusion restriction on DWI/ADC. Gadolinium enhancement was heterogeneous and avid, with small nonenhancing foci. No other enhancing intracranial lesion was identified (**Figure 1**). Initial thoracic CT revealed no suspicious pulmonary lesion, hilar or mediastinal lymphadenopathy, pleural abnormality, or thoracic mass (**Figure 2**). Whole-body FDG PET/CT and serial thoracic CT were not performed; systemic staging therefore remained incomplete.

**Figure 1:**
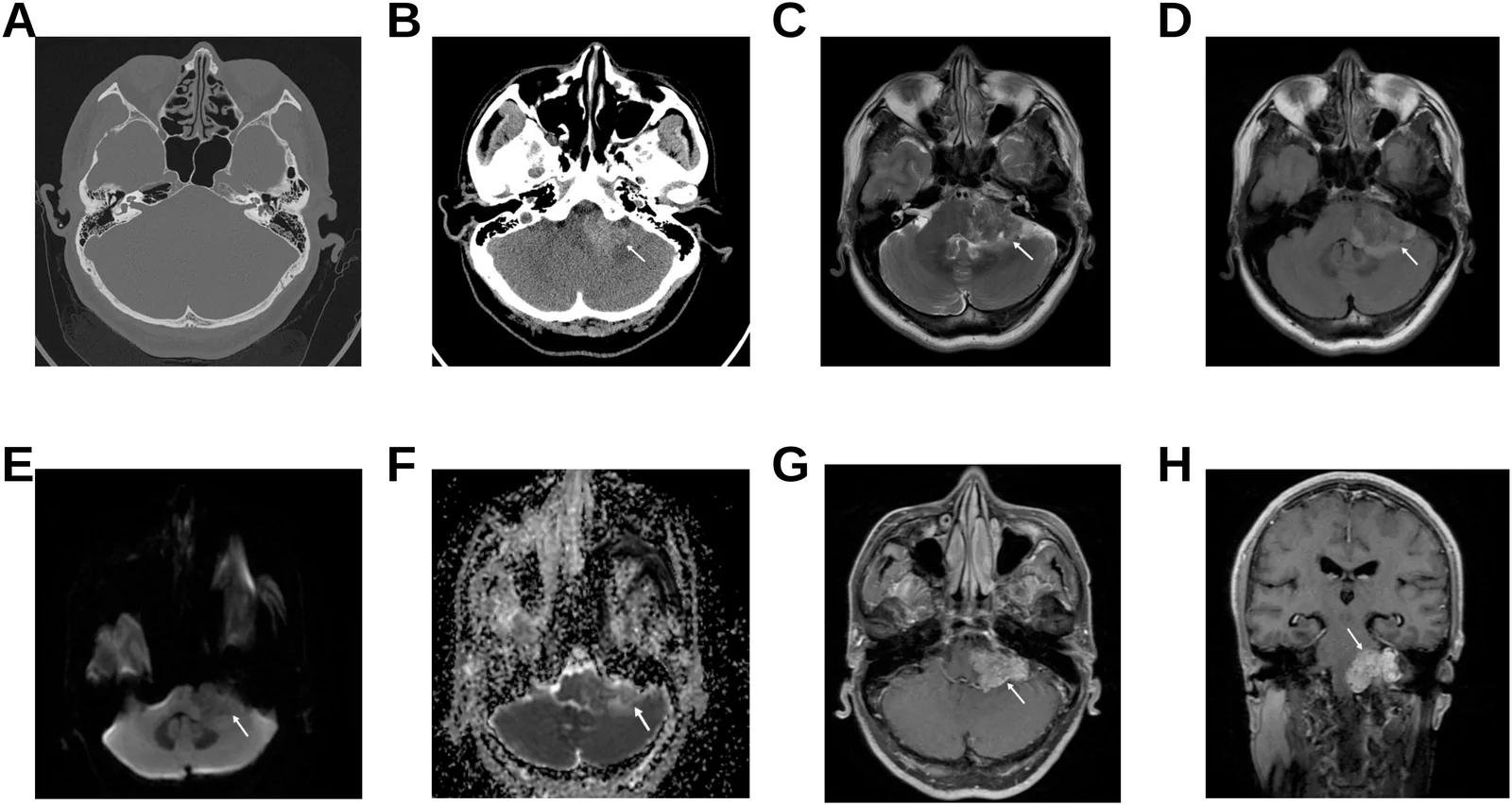
Neuroimaging of the left CPA-region lesion. **(A)** Axial bone-window CT shows no obvious adjacent petrous bone destruction or IAC enlargement. **(B)** Non-contrast CT shows a slightly hyperdense CPA-region mass. **(C)** T2-weighted MRI shows predominantly isointense signal with internal linear mildly hyperintense components. **(D)** FLAIR shows mixed signal and mild adjacent edema. **(E, F)** DWI and ADC show no definite diffusion restriction. **(G, H)** Axial and coronal contrast-enhanced T1-weighted images show heterogeneous avid enhancement with small nonenhancing foci. The lesion measured approximately 37.6 × 24.7 × 35.9 mm (left-to-right [transverse] × anteroposterior × craniocaudal) on contrast-enhanced T1-weighted MRI; the left-to-right and anteroposterior dimensions were measured on axial images, and the craniocaudal dimension was measured on a sagittal image not shown. The lesion compressed the brainstem, cerebellum, and fourth ventricle. Brainstem invasion was suspected, not established; the extra-axial-like appearance did not securely establish a purely extra-axial compartment. Arrows indicate the lesion.

**Figure 2:**
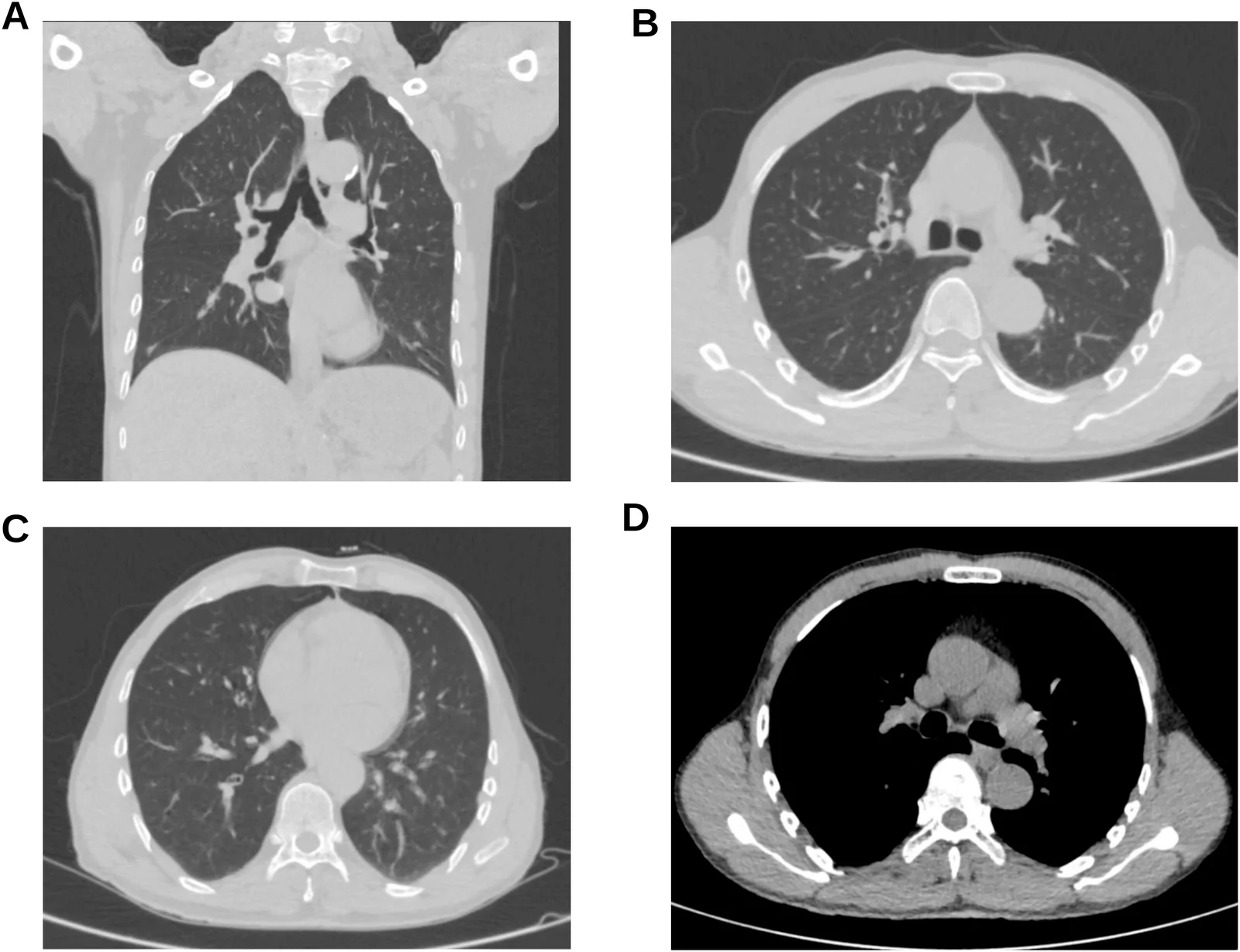
Initial thoracic CT without an identifiable pulmonary primary. **(A)** Coronal and **(B, C)** axial lung-window images show no identifiable pulmonary mass, suspicious nodule, ground-glass lesion, consolidation, or obstructive atelectasis. **(D)** Mediastinal-window CT shows no hilar or mediastinal lymphadenopathy, pleural abnormality, or thoracic mass. This was an initial examination; no serial thoracic CT or whole-body FDG PET/CT was performed.

#### 2.2.2 Imaging differential diagnosis

Vestibular schwannoma was plausible given hearing loss and dizziness, but absent IAC enlargement or a definite intracanalicular component was atypical. Meningioma was considered because of location and enhancement, although no broad dural attachment, dural tail, calcification, or hyperostotic response was demonstrated. Avid enhancement and absent diffusion restriction argued against an epidermoid cyst. The short symptom history, heterogeneous enhancement, edema, and suspected brainstem involvement prompted consideration of metastasis. These findings overlap between entities and are not individually diagnostic. **Supplementary Table S2** compares IAC and dural relationships, bone response, diffusion, enhancement, edema, compartment, and clinical tempo (5–7).

#### 2.2.3 Histopathology and pathological differential diagnosis

Two pathologists reviewed the available slides. Histology demonstrated complex glandular and papillary epithelial architecture, clear to eosinophilic cytoplasm with prominent supranuclear and subnuclear vacuoles, high-grade atypia, and patchy necrosis. Immunohistochemistry showed diffuse strong pan-cytokeratin, focal CK7, focal nuclear TTF-1 and SALL4, partial synaptophysin, and focal weak CD56 positivity. Ki-67 was approximately 80% in a proliferative hotspot (**Supplementary Figure S1**). CK20, SATB2, PAX8, PAX2, NKX3.1, GFAP, Napsin A, S100, SOX10, OCT3/4, and CD117 were negative. Representative findings are shown in **Figure 3**.

**Figure 3:**
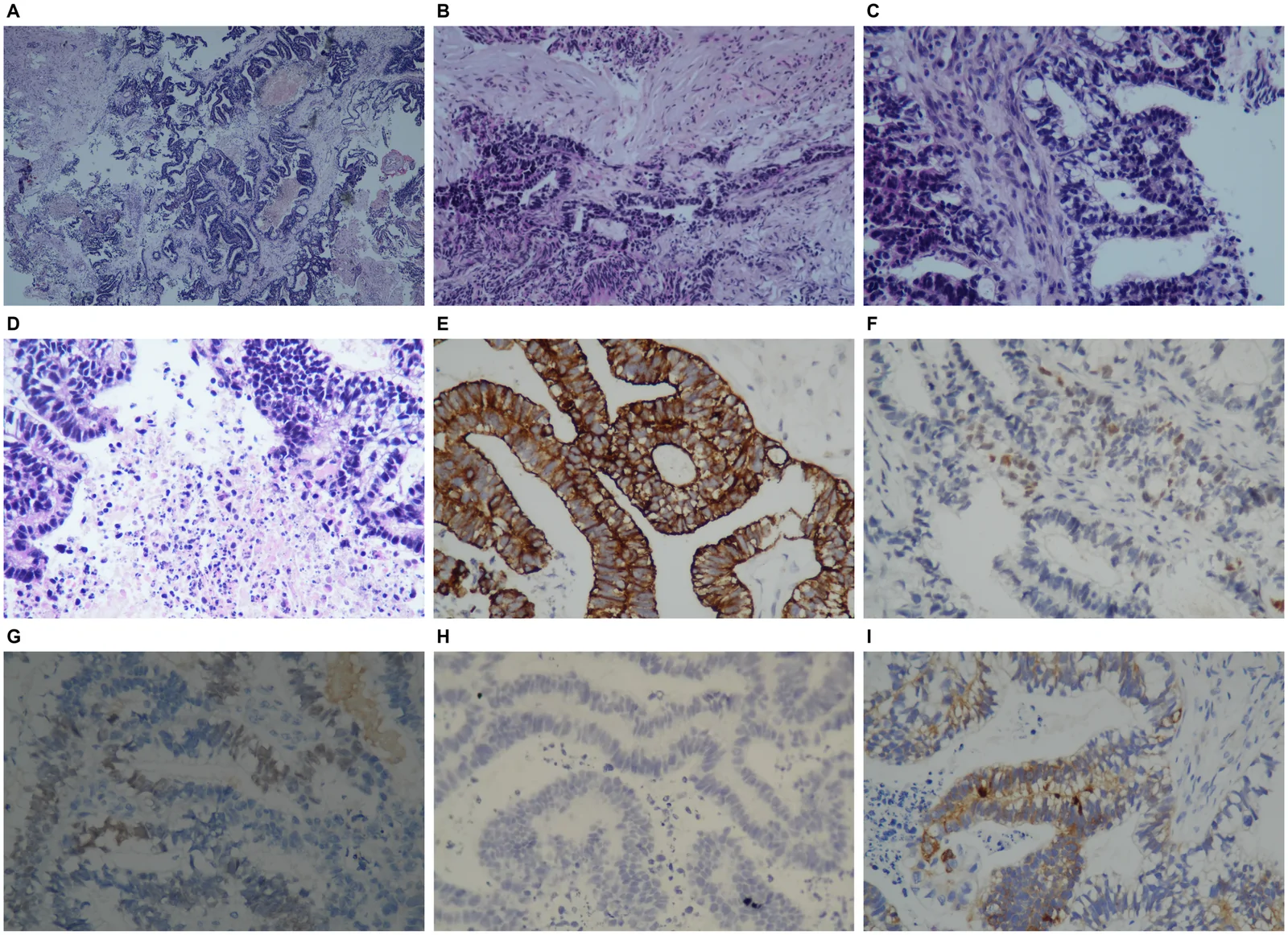
Histopathological and immunohistochemical findings of the CPA-region tumor. **(A)** H&E shows complex glandular and papillary architecture (original magnification, ×40). **(B)** H&E demonstrates fetal-type glandular structures (×200). **(C)** H&E shows clear to eosinophilic tumor cells with supranuclear and subnuclear vacuoles and high-grade atypia (×400). **(D)** H&E demonstrates tumor necrosis adjacent to viable tissue (×400). **(E)** Pan-cytokeratin shows diffuse strong tumor-cell positivity (×400). **(F)** TTF-1 shows focal nuclear positivity (×400). **(G)** SALL4 shows focal nuclear positivity (×400). **(H)** Napsin A is negative in tumor cells (×400). **(I)** Synaptophysin shows partial cytoplasmic positivity (×400). The integrated interpretation was metastatic adenocarcinoma with high-grade fetal morphology, favoring pulmonary origin; a pulmonary primary was neither identified on initial thoracic CT nor confirmed pathologically, and pulmonary HG-FLAC was not established. Ki-67 is shown in **Supplementary Figure S1**.

Focal TTF-1 favored pulmonary differentiation but did not establish pulmonary origin. Negative Napsin A neither excluded lung adenocarcinoma nor provided positive evidence of a pulmonary primary. Focal CK7 supported an epithelial phenotype but was not site-specific. SALL4 indicated an oncofetal phenotype without identifying a primary site. Partial synaptophysin and focal weak CD56 expression, interpreted with dominant gland-forming epithelial architecture, did not by themselves establish a primary CNS or neuroendocrine neoplasm (2–4, 8).

Adult CPA medulloblastoma was considered, but the tumor lacked predominantly primitive small-cell/neuroblastic morphology and instead showed complex epithelial glands and diffuse keratin expression (9). Characteristic rhabdoid morphology was not identified to favor atypical teratoid/rhabdoid tumor (AT/RT); however, INI1/SMARCB1 and BRG1/SMARCA4 assessment was unavailable (10). Embryonal tumor with multilayered rosettes (ETMR), including rare adult CPA/IAC presentations, was less likely because of age and absent multilayered rosettes or neuropil-rich embryonal architecture. LIN28A and C19MC/DICER1 testing were not performed (11, 12). Negative GFAP and dominant epithelial gland formation argued against conventional glial/ependymal differentiation but could not exclude every variant.

Choroid plexus tumors, particularly carcinoma, also merit consideration because papillary architecture and keratin expression can overlap with metastatic adenocarcinoma. A choroid plexus origin was not established anatomically, and GFAP/S100 negativity was not sufficient for exclusion. Kir7.1, stanniocalcin-1, and transthyretin were not assessed; this differential therefore remains incompletely resolved (13).

Yolk sac and hepatoid tumors were also considered. SALL4 positivity can occur in yolk sac tumors despite OCT3/4 negativity; negative OCT3/4 and CD117 were not treated as exclusionary (14). Normal serum AFP did not replace tissue assessment. Tissue AFP, glypican-3, beta-catenin, HepPar-1, and arginase-1 were not tested. Remaining paraffin-embedded tissue was insufficient for additional immunohistochemistry.

**Supplementary Table S1** sets out the morphological overlap, opposing findings, available IHC, missing ancillary studies, and residual uncertainty for each major alternative. A primary CNS or another metastatic origin was considered less likely in the integrated interpretation, but not definitively excluded.

#### 2.2.4 Integrated diagnosis

Metastatic adenocarcinoma with high-grade fetal morphology, favoring pulmonary origin; however, no pulmonary primary was identifiable on initial thoracic CT or confirmed pathologically. The diagnosis of pulmonary HG-FLAC was therefore not established.

The diagnosis rested principally on fetal-type morphology and epithelial differentiation, with focal TTF-1 providing limited support for pulmonary origin. Negative initial thoracic CT, incomplete systemic staging, and unavailable ancillary studies prevent a more definitive primary-site designation.

#### 2.2.5 Therapeutic intervention

A retrosigmoid approach was used. The tumor was firm and hypervascular, with dense adhesion to the facial-vestibulocochlear complex, lower cranial nerves, and brainstem. After opening the capsule and mapping nerve courses electrophysiologically, the tumor was removed piecemeal. A small residual component was intentionally left to avoid injury, resulting in subtotal resection. Adhesion alone did not establish histological brainstem invasion.

After tertiary-center consultation, no systemic anticancer therapy or radiotherapy was initiated, and the patient received best supportive care at a local hospital. Available records did not provide a postoperative ECOG/KPS score, formal multidisciplinary recommendations, a documented stereotactic or conventional radiotherapy assessment, systemic-treatment eligibility or tolerance assessment, or a clear account of patient/family preference or refusal. Postoperative brain MRI/CT findings sufficient to quantify residual disease or assess progression were unavailable; absence of retrievable imaging cannot establish that no postoperative scan occurred. The operative record established residual tumor. These missing details prevent reconstruction of the oncological decision pathway and must not be interpreted as a deliberate management strategy.

#### 2.2.6 Follow-up and outcomes

Following postoperative intensive care and transfer to the ward, dysphagia with coughing or choking on liquids and facial nerve dysfunction with impaired cheek puffing and lagophthalmos developed. These deficits were anatomically compatible with facial and lower cranial nerve-related dysfunction in the context of the operative findings. Their severity was not documented with standardized functional scales.

The patient died 3 months after surgery. The immediate cause of death was undocumented. Residual tumor, postoperative swallowing impairment, incomplete staging, and lack of tumor-directed therapy confounded the outcome. The records could not distinguish progression, aspiration-related complications, other postoperative morbidity, or another cause of death. This survival interval cannot be used as evidence of HG-FLAC-specific prognosis.

## 3 Discussion

The educational value is the discordance between an extra-axial-like CPA presentation and an epithelial malignancy with high-grade fetal morphology, despite negative initial thoracic CT. Pulmonary HG-FLAC can exhibit heterogeneous differentiation and molecular findings, and early relapse has been reported in confirmed pulmonary cases (15, 16). Those observations provide context rather than proof of the primary site or natural history in this patient. The term metastatic adenocarcinoma with high-grade fetal morphology, favoring pulmonary origin is used throughout; pulmonary HG-FLAC was not established.

Immunophenotypic overlap requires that morphology, anatomical context, and the limitations of each stain be weighed together. CK7 and TTF-1 supported the favored interpretation only within this context; SALL4 and neuroendocrine marker expression could not establish a site. The comparative assessment in **Supplementary Table S1** explains why several alternatives were less favored without equating missing tests with negative results. Supplemental testing would have been valuable, but remaining tissue was insufficient for additional immunohistochemistry.

EGFR, ALK, ROS1, BRAF, KRAS, MET, RET, and NTRK testing and, separately, PD-L1 immunohistochemistry were not performed. A reported pulmonary HG-FLAC with MET amplification and brain metastasis responded to savolitinib (17); another report illustrated substantial diagnostic mimicry (18). MET amplification and MET exon 14 skipping are distinct abnormalities with different therapeutic evidence. An actionable MET finding might have informed consideration of MET-directed therapy, but neither alteration would establish pulmonary origin, and the cited HG-FLAC case concerned amplification, not exon 14 skipping. PD-L1 assessment could have informed systemic-treatment discussions but would neither identify the primary site nor guarantee benefit.

Ancillary studies also could have changed the differential diagnosis: C19MC amplification in an appropriate embryonal tumor would favor ETMR, while SMARCB1/INI1 or SMARCA4/BRG1 loss would prompt evaluation for AT/RT in the relevant morphology. Neither protein loss nor a molecular result should be interpreted independently of the pathological context (10–12). Thus, missing studies limited both diagnostic confidence and assessment of treatment options; they cannot retrospectively determine which treatment this patient would have tolerated or benefited from.

### 3.1 Strengths and limitations

The principal strength is direct imaging-pathology correlation in an unusual CPA presentation. Limitations include no whole-body FDG PET/CT or serial thoracic CT, no demonstrated pulmonary primary, and no genomic profiling or PD-L1 assessment. INI1/SMARCB1, BRG1/SMARCA4, LIN28A, beta-catenin, tissue AFP, glypican-3, HepPar-1, arginase-1, and choroid plexus markers were not assessed; C19MC/DICER1 analysis was not performed. Insufficient remaining paraffin-embedded tissue prevented additional immunohistochemical work-up. CK7, CD56, and Ki-67 had already been performed and are not missing tests. Incomplete postoperative imaging, functional assessments and oncological decision records, together with an undocumented immediate cause of death, further constrain interpretation. The case cannot establish a persistently occult lung primary, fully resolve all alternative lineages, or estimate subtype-specific survival.

## 4 Conclusion

Metastasis should remain in the differential for an atypical CPA mass despite negative initial thoracic CT. This case supports a diagnosis of metastatic adenocarcinoma with high-grade fetal morphology, favoring pulmonary origin; no pulmonary primary was identified on initial thoracic CT or confirmed pathologically, and pulmonary HG-FLAC was not established. Complete staging and appropriately selected ancillary studies are important when feasible. Brainstem invasion remained suspected, and the fatal outcome could not be assigned to a specific cause.

## 5 Patient perspective

A patient-authored account was unavailable because of postoperative neurological deficits and death during follow-up. Family members described initially understanding the illness as a brain lesion causing imbalance, dizziness, and hearing difficulty. The interpretation of metastatic adenocarcinoma with high-grade fetal morphology, favoring pulmonary origin, was unexpected after negative chest CT. According to the family, tumor-directed treatment was not pursued after tertiary consultation, and supportive care followed; a documented reason for that decision was unavailable.

## Data Availability

The data analyzed in this study is subject to the following licenses/restrictions: The underlying original clinical records, source imaging data, and pathological records are not publicly available because they may contain potentially identifiable or protected patient information and are subject to institutional privacy restrictions. Additional de-identified information may be made available by the corresponding author upon reasonable request, subject to appropriate institutional approval and applicable patient-privacy and consent requirements. Requests to access these datasets should be directed to CY, a15698122515@163.com.

## Supplementary material

Supplementary Figure 1 Shows Ki-67 immunohistochemistry.

Supplementary Table 1 Presents the pathological differential diagnosis.

Supplementary Table 2 Compares the imaging features of CPA lesions.
