# Supplementary material for: Case Report: Cerebellopontine angle metastatic adenocarcinoma with high-grade fetal morphology and no identifiable pulmonary primary on initial CT
Source: Front Oncol. 2026 Oct 27;16:1942218. doi: 10.3389/fonc.2026.1942218 (PMC13747754; doi:10.3389/fonc.2026.1942218)
Supplement: Supplementary file 1 [file Supplementaryfile1.docx]

Supplementary Figure 1

Ki-67 immunohistochemistry of the CPA-region tumor. A representative proliferative hotspot shows extensive tumor-cell nuclear staining; the reported Ki-67 proliferation index was approximately 80% (original magnification, ×400). This finding supports high proliferative activity but does not establish the primary site.

# Supplementary Table S1

Differential diagnosis of primary CNS tumors and other tumors with fetal, hepatoid, or glandular morphology.

| **Diagnostic consideration** | **Overlapping/supportive features** | **Findings against or limiting support** | **Relevant IHC performed in this case** | **Additional studies not performed** | **Assessment in this case** |
| --- | --- | --- | --- | --- | --- |
| Adult CPA medulloblastoma | Rare adult CPA presentation; high-grade cytology and synaptophysin expression can overlap. | No predominantly primitive small-cell/neuroblastic pattern; complex epithelial glands dominate. | Diffuse pan-CK; partial synaptophysin; focal weak CD56. | Molecular subgrouping or methylation profiling if morphology warranted. | Less favored morphologically; no molecular exclusion. [1] |
| AT/RT | Can show epithelial and neuroendocrine differentiation, including in adults. | Characteristic rhabdoid morphology not identified; fetal-type glands dominate. | Pan-CK positive; synaptophysin partial; GFAP negative. | INI1/SMARCB1 and BRG1/SMARCA4 IHC; corresponding molecular studies if indicated. | Not definitively excluded. Protein loss requires integrated interpretation. [2] |
| ETMR | Embryonal morphology can overlap; rare adult IAC/CPA cases exist. | Age 61; no multilayered rosettes or neuropil-rich embryonal architecture identified. | Pan-CK positive; partial synaptophysin; GFAP negative; profile not diagnostic. | LIN28A IHC; C19MC amplification/DICER1 analysis and molecular classification if indicated. | Substantially less likely; not excluded by ancillary testing. [3, 4] |
| Glial/ependymal neoplasm | Posterior fossa origin and apparent CPA extension can overlap. | Dominant epithelial gland formation and GFAP negativity do not favor conventional glial/ependymal differentiation. | GFAP negative; pan-CK diffuse positive. | EMA pattern and lineage-directed IHC; selected molecular classification if morphology warranted. | Less favored; GFAP alone cannot exclude all variants. |
| Choroid plexus tumor, especially carcinoma | Papillary epithelial architecture, keratin expression, and CPA/fourth-ventricular proximity overlap. | A choroid plexus origin was not demonstrated; current markers cannot resolve this alternative. | Pan-CK positive; focal CK7; CK20, GFAP and S100 negative. | Kir7.1, stanniocalcin-1, transthyretin; further classification if indicated. | Incompletely excluded. Negative GFAP/S100 is insufficient. [5] |
| Yolk sac tumor or yolk sac differentiation | Fetal/glandular morphology, epithelial differentiation and SALL4 expression overlap. | Only focal SALL4; serum AFP normal, but neither finding is exclusionary. | SALL4 focal positive; pan-CK positive; OCT3/4 and CD117 negative. | Tissue AFP and glypican-3; targeted clinical/systemic evaluation. | Not fully excluded. OCT3/4/CD117 negativity does not exclude yolk sac differentiation. [6] |
| Hepatoid adenocarcinoma or another hepatocellular-type metastasis | Eosinophilic/clear cytoplasm, fetal-type differentiation and oncofetal marker expression can overlap. | Glandular/papillary phenotype predominates; a hepatic or other systemic primary was not established. | Pan-CK positive; focal SALL4/CK7; serum AFP normal (not an IHC result). | HepPar-1, arginase-1, tissue AFP, glypican-3; site-directed evaluation. | Not fully excluded; no dedicated tissue panel and incomplete staging. |
| Metastatic pulmonary HG-FLAC | Fetal-type glands/vacuoles, high-grade atypia, necrosis and focal TTF-1 support consideration. | No pulmonary primary on initial CT or pathology; focal TTF-1 and CK7 are not site-specific; Napsin A negative. | Pan-CK diffuse; TTF-1/CK7/SALL4 focal; partial synaptophysin; weak focal CD56. | Beta-catenin/oncofetal studies; genomic profiling and PD-L1 for characterization/treatment, not proof of origin. | Pulmonary origin favored, unconfirmed. Pulmonary HG-FLAC not established. [7–9] |
| Other metastatic fetal/clear-cell/glandular tumors | Clear/eosinophilic cytoplasm and gland formation are shared by multiple carcinomas. | Available site-associated markers make selected colorectal, renal and prostatic lineages less likely, not impossible. | CK20/SATB2, PAX8/PAX2 and NKX3.1 negative; focal CK7/TTF-1. | Site-directed ancillary markers selected by morphology and clinical findings; complete systemic evaluation. | No exhaustive exclusion or definite primary-site assignment. |
| Neuroendocrine neoplasm | Synaptophysin and CD56 expression; high Ki-67. | Predominantly complex gland-forming epithelium; limited marker expression alone is insufficient for classification. | Synaptophysin partial; CD56 focal weak; pan-CK diffuse; Ki-67 approximately 80%. | Chromogranin A/INSM1 if morphology warrants; relevant site/lineage work-up. | Marker expression interpreted as possible divergent differentiation; primary CNS/neuroendocrine origin not established. [7] |

The assessment summarizes available morphology and reported IHC, not an exhaustive exclusion panel. Studies in the “not performed” column are potential discriminators selected according to morphology; they are not universally mandatory. Remaining paraffin-embedded tissue was insufficient for additional IHC. CK7, CD56 and Ki-67 were already performed. No primary CNS or other systemic lineage is declared definitively excluded.

# Supplementary Table S2

Imaging differential diagnosis of cerebellopontine angle lesions.

| **Feature** | **Vestibular schwannoma** | **Meningioma** | **Epidermoid cyst** | **CPA metastasis** | **Present case** |
| --- | --- | --- | --- | --- | --- |
| IAC involvement | Often an intracanalicular component and smooth widening. | May enter IAC; widening less characteristic. | Usually cisternal; no characteristic IAC widening. | Variable; may involve IAC or cranial nerves without widening. | No definite IAC enlargement or intracanalicular extension. |
| Dural relationship | No characteristic broad dural attachment or tail. | Broad attachment and dural tail often present. | No characteristic dural attachment. | Variable; dural metastases can mimic meningioma. | No definite broad attachment or dural tail. |
| Bone response | Smooth canal remodeling may occur. | Hyperostosis/calcification may occur; absence does not exclude. | Usually no characteristic bone response. | Variable; destruction possible, but may be absent. | No definite petrous erosion, hyperostosis, or calcification established. |
| DWI/ADC | Variable; restricted diffusion is not a defining feature. | Variable; some tumors show lower ADC. | Typically conspicuous DWI signal/restricted diffusion relative to CSF. | Variable with tumor type/cellularity; absence of restriction does not exclude. | No definite diffusion restriction on DWI/ADC. |
| Enhancement | Avid; may be heterogeneous/cystic in larger lesions. | Often avid and relatively homogeneous; variants occur. | Usually no enhancement. | Often avid, heterogeneous or ring-like; variable. | Heterogeneous avid enhancement with small nonenhancing foci. |
| Adjacent edema | Variable, especially with larger masses. | Variable; can be present. | Usually little or no edema. | Common but variable; may be mild. | Mild adjacent edema. |
| Compartment and interface | Usually extra-axial, with displacement/compression. | Usually dural-based extra-axial; invasion can occur. | Extra-axial/cisternal, insinuating around structures. | May be parenchymal, meningeal or extra-axial-like. | CPA/cisternal, extra-axial-like; brainstem invasion suspected, not proven. Purely extra-axial compartment uncertain. |
| Clinical tempo | Often gradual; variability exists. | Often gradual; variability exists. | Usually slow; delayed symptoms possible. | Can progress rapidly; clinical tempo is not diagnostic. | Gait instability for >1 month, dizziness and hearing loss; serial tumor growth not measured. |

These are characteristic patterns with substantial overlap, not diagnostic rules. The present case measured 37.6 × 24.7 × 35.9 mm, with brainstem/cerebellar mass effect and fourth-ventricle compression. Close relationships with cranial nerves VII–VIII and lower cranial nerves were documented mainly at surgery. Specific vermian involvement and the exact cisternal site of origin were not established. Sources: [10–12].

# Supplementary references

1. Pant I, Chaturvedi S, Gautam VKS, Sarma P, Satti DK. Extra-axial adult cerebellopontine angle medulloblastoma: revisiting a rare entity. J Cancer Res Ther. 2022;18(3):770–773. doi:10.4103/jcrt.JCRT_675_20.

2. Makuria AT, Rushing EJ, McGrail KM, Hartmann DP, Azumi N, Ozdemirli M. Atypical teratoid rhabdoid tumor (AT/RT) in adults: review of four cases. J Neurooncol. 2008;88(3):321–330. doi:10.1007/s11060-008-9571-z.

3. Chadda KR, Solano-Páez P, Khan S, Llempén-López M, Phyu P, Horan G, et al. Embryonal tumor with multilayered rosettes: overview of diagnosis and therapy. Neurooncol Adv. 2023;5(1):vdad052. doi:10.1093/noajnl/vdad052.

4. Sheriff A, Takami H, Takayanagi S, Kitagawa Y, Tanaka S, Ikemura M, et al. Embryonal tumor with multilayered rosettes arising from the internal auditory canal of an adult: illustrative case with molecular investigations. Neuropathology. 2024;44(3):208–215. doi:10.1111/neup.12951.

5. Hasselblatt M, Böhm C, Tatenhorst L, Dinh V, Newrzella D, Keyvani K, et al. Identification of novel diagnostic markers for choroid plexus tumors: a microarray-based approach. Am J Surg Pathol. 2006;30(1):66–74. doi:10.1097/01.pas.0000176430.88702.e0.

6. Wang F, Liu A, Peng Y, Rakheja D, Wei L, Xue D, et al. Diagnostic utility of SALL4 in extragonadal yolk sac tumors: an immunohistochemical study of 59 cases with comparison to placental-like alkaline phosphatase, alpha-fetoprotein, and glypican-3. Am J Surg Pathol. 2009;33(10):1529–1539. doi:10.1097/PAS.0b013e3181ad25d5.

7. Suzuki M, Yazawa T, Ota S, Morimoto J, Yoshino I, Yamanaka S, et al. High-grade fetal adenocarcinoma of the lung is a tumour with a fetal phenotype that shows diverse differentiation, including high-grade neuroendocrine carcinoma: a clinicopathological, immunohistochemical and mutational study of 20 cases. Histopathology. 2015;67(6):806–816. doi:10.1111/his.12711.

8. Li Y, Xi SY, Yong JJ, Wu XY, Yang XH, Wang F. Morphologic, immunohistochemical, and genetic differences between high-grade and low-grade fetal adenocarcinomas of the lung. Am J Surg Pathol. 2021;45(11):1464–1475. doi:10.1097/PAS.0000000000001744.

9. Ye J, Findeis-Hosey JJ, Yang Q, McMahon LA, Yao JL, Li F, et al. Combination of napsin A and TTF-1 immunohistochemistry helps in differentiating primary lung adenocarcinoma from metastatic carcinoma in the lung. Appl Immunohistochem Mol Morphol. 2011;19(4):313–317. doi:10.1097/PAI.0b013e318205b059.

10. Yuh WT, Mayr-Yuh NA, Koci TM, Simon JH, Nelson KL, Zyroff J, et al. Metastatic lesions involving the cerebellopontine angle. AJNR Am J Neuroradiol. 1993;14(1):99–106.

11. Minami M, Hanakita J, Suwa H, Suzui H, Fujita K, Nakamura T. Solitary metastasis of lung cancer to the cerebellopontine angle: case report. Neurol Med Chir (Tokyo). 1996;36(3):172–174. doi:10.2176/nmc.36.172.

12. Lal S, Chaturvedi S, Pant I. Historadiological correlation of cerebellopontine angle tumors: series of 122 cases. Indian J Otolaryngol Head Neck Surg. 2021;73(1):45–51. doi:10.1007/s12070-020-02022-5.

## Abbreviations

ADC, apparent diffusion coefficient; AFP, alpha-fetoprotein; AT/RT, atypical teratoid/rhabdoid tumor; CK, cytokeratin; CNS, central nervous system; CPA, cerebellopontine angle; DWI, diffusion-weighted imaging; EMA, epithelial membrane antigen; ETMR, embryonal tumor with multilayered rosettes; GFAP, glial fibrillary acidic protein; HG-FLAC, high-grade fetal adenocarcinoma of the lung; IAC, internal auditory canal; IHC, immunohistochemistry; PD-L1, programmed death-ligand 1; TTF-1, thyroid transcription factor-1.
